# Aromatic Volatile Substances in Different Types of Guangnan Dixu Tea Based on HS-SPME-GC-MS Odor Activity Value

**DOI:** 10.3390/metabo15040257

**Published:** 2025-04-09

**Authors:** Ying Feng, Di Tian, Chaoliang Wang, Yong Huang, Yang Luo, Xiuqiong Zhang, Lei Li

**Affiliations:** 1 College of Sanqi Medical, Wenshan College, Wenshan 663099, China; fy1085040603@outlook.com (Y.F.); 17869186521@163.com (C.W.); huangyong-1159@163.com (Y.H.); unicorn19@163.com (Y.L.); 2 College of Food Science and Technology, Yunnan Agricultural University, Kunming 650051, China; m17853435722@163.com; 3 College of Chemistry and Chemical Engineering, Yunnan Normal University, Kunming 650500, China

**Keywords:** HS-SPME-GC-MS, Guangnan Dixu tea, aroma components, OAV

## Abstract

Dixu tea is one of the characteristic tea germplasm resources of southeastern Yunnan, and is also a precious wild tea germplasm resource. **Background**: In order to further develop Dixu tea products and improve their flavor, this article studies the effects of different processing methods on the aroma quality of Dixu tea. **Methods**: A comprehensive analysis of the aroma quality of Diwei tea was conducted using HS-SPME combined with GC-MS and multivariate statistical analysis. A principal component analysis (PCA) was applied to process the detected volatile substances and an orthogonal partial least squares-discriminant analysis (OPLS-DA) model was established. We evaluated the contribution of major compounds in the tea aroma by calculating the odor activity value (OAV). **Results**: The results showed that a total of 67 compounds were identified. A total of 27 major aromatic volatile compounds (OAV > 1) were screened, and 17 key differential volatile compounds were identified in different tea samples, including octanoic acid, d-citrol, laurene, hexanal, citral, β-cyclic citral, trans-2-hexenal, γ-nonanolide, β-ionone, geranylacetone, 1,1,6-trimethyl-1,2-dihydronaphthalene, geraniol, methyl salicylate, linalool, nerolidol, and 7,11-dimethyl-3-methylene-1,6,10-dodecatriene. Combined with the OAV analysis, it is shown that a floral fragrance is a common feature of Guangnan Dixu tea varieties. In addition, white tea also has a fragrant aroma, while black tea, green tea, and bamboo tube tea are all accompanied by a fruity aroma. **Conclusions**: In summary, processing techniques regulate the aroma characteristics of various types of tea by changing the types and contents of volatile aroma compounds. This provides a theoretical basis for exploring and utilizing tea production resources in the future.

## 1. Introduction

Tea is one of the most widely consumed non-alcoholic beverages worldwide, known for its anti-cancer, anti-bacterial, and anti-inflammatory properties [1]. Based on the processing methods, tea can be categorized into six types: green tea, white tea, yellow tea, oolong tea, black tea, and dark tea.

Volatile compounds represent approximately 0.01% to 0.05% of the total dry matter in tea, serving as a crucial indicator for evaluating tea quality [2]. Different teas exhibit distinct aroma characteristics [3,4], which arise from variations in the raw material processing methods, types of tea, and harvesting seasons. Zhang Xuebo et al. [5] observed that, while the volatile compositions of tea samples made using various processing methods were largely similar, the relative content of each component varied significantly. For instance, Xie Guanhua et al. [6] discovered that the aroma differences among six categories of tea resulted from varying proportions of key aroma compounds, which are crucial for defining tea quality. Tang Rong et al. [7], focusing on the ancient tea tree group in Xinping County, investigated local tea-making processes of producing green tea, black tea, white tea, yellow tea, and ripe tea. Through the analysis of the biochemical composition and volatile aroma components, it was found that out of the ancient tea leaves, white tea demonstrated superior quality and suitability [8].

According to the Guangnan County Annals, Dixu tea has a history of over 300 years, named after its origin in Dixu Township. In 2016, the former Ministry of Agriculture of the People’s Republic of China officially granted registration and protection for the geographical indication of “Dixu Tea”. Dixu tea is a unique local tea germplasm resource found in Southeast Yunnan and represents a rare example of wild tea germplasm. It not only demonstrates the continuity of tea germplasm resources in Southeast Yunnan but also exhibits shared traits with the germplasm resources of tea trees in Yunnan and Guangxi. The youthful and unique aroma of the tea, combined with excellent varieties and a distinct climate, contributes to its exceptional quality. When brewed as green tea, it produces an infusion characterized by a bright green color and a sweet aftertaste. This tea is also highly regarded in the market and serves as a primary raw material for Pu-Erh tea [9]. Among these varieties, zhutong tea is a local flavor tea of Yunnan’s ethnic minorities [10]. Known as “girl tea” and “Wazana” in Chinese due to its ripe raw materials, zhutong tea is produced in Menghai and Guangnan Counties, located in Wenshan Prefecture, Xishuangbanna, Yunnan. It predominantly uses large leaf seeds to produce dried green tea and is considered one of Yunnan’s most ancient and renowned tea varieties. Zheng Lin et al. [11] conducted a chemical composition analysis of bamboo tube tea using steam distillation extraction (SDE) and gas chromatography-mass spectrometry (GC-MS). They concluded that the main volatile components included physosol, palmitic acid, linalool, phenylacetaldehyde, ethyl palmitate, and ethyl linoleate.

Currently, research on Dixu tea is limited to the discrimination of the aroma of the air-dried tea leaves and the extraction of flavonoids from its tea leaves. Si, H.Y.; Jiu, M.L.; et al. [12,13,14] have investigated green tea, black tea, and Pu-Erh tea; however, studies on the aroma quality of the different tea varieties derived from Guangnan Dixu tea are limited. Therefore, this study employs headspace solid-phase micro-extraction gas chromatography-mass spectrometry (HS-SPME-GC-MS), a solvent-free, simple, and sensitive sample pretreatment method that selectively adsorbs volatile or semi-volatile compounds, making it suitable for the analysis of aroma components in complex matrices such as tea and food. GC-MS (gas chromatography-mass spectrometry) separates and identifies compounds, providing an efficient platform for the qualitative and quantitative analysis of aroma substances. In this study, this method was utilized to extract and analyze volatile compounds in class 4 tea to determine the main components affecting its aroma. The aim of this study is to reveal the aroma quality characteristics of Dixu tea and explore the differences in major aroma compositions among its various tea types. This research provides a theoretical foundation for the sustainable utilization of tea production resources, the development of high-quality tea, the enhancement of Dixu tea varieties, and the utilization of diverse tea types in the future.

## 2. Materials and Methods

### 2.1. Preparation of Tea Samples

**Figure 1:**
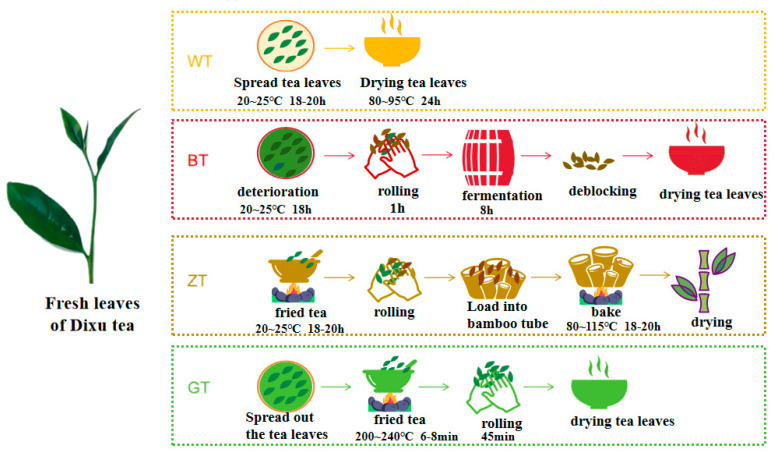
Processing steps of 4 types of Dixu tea.

In this study, fresh leaves from Diyi Village, Guangnan, grown under consistent environmental conditions and free from pests and diseases, were selected and processed into white tea (WT), black tea (BT), zhutong tea (ZT), and green tea (GT) using traditional processing methods (Figure 1).

In this study, fresh tea leaves from Guangnan Dixu, grown under consistent environmental conditions and free of pests and diseases, were used. The fresh leaves were spread out and left to wither at 20–25 °C for 18–20 h, followed by drying at 80–95 °C to produce white tea (WT). For black tea (BT), the fresh leaves were withered for 18 h, rolled for 1 h, fermented for 8 h, and then dried. Bamboo tube tea (ZT) was produced by first subjecting the fresh leaves to fixation (de-enzyming), then rolling them, placing them into bamboo tubes, and roasting them over a charcoal fire for 18 h before drying. Green tea (GT) was produced by fixation of the fresh leaves, followed by rolling and drying (Figure 1). Three biological replicates were utilized in the experiment.

### 2.2. Experimental Equipment

Tea integrated processing equipment: 6 CZ-110I (Fujian Wangshu Tea Machinery, Wuyishan, China); tea rolling machine: 6CR-55 (Zhejiang Gashan Industry, Shengzhou, China); breaking machine: 6 C3 (Fujian Clover Tea Machinery, Quanzhou, China); frying machine: 6 CWC-100 (Fujian Jiayu Machinery, Sanming, China); dryer: JY-6 CHZ-7B (Fujian Jiayu Machinery, Sanming, China). DVB/CAR/PDMS solid-phase microextraction head (50/30 μm, Merck, Rahway, NJ, USA); PDMS/DVB extraction needle (Supelco, Bellefonte, PA, USA); 7890B (Agilent Technologies, Santa Clara, CA, USA).

### 2.3. Extraction and Analysis of Volatile Compounds

#### 2.3.1. Quantitative Descriptive Analysis

Quantitative Descriptive Analysis (QDA) is a widely employed method for evaluating the characteristic attributes of samples. It provides a more accurate and intuitive description of sensory properties, making it a comprehensive method for quantifying sensory evaluation results. This method has been widely applied in the sensory evaluation of various tea samples [15]. To conduct a more comprehensive and scientific analysis of the samples’ aroma, the specific aroma characteristics were further evaluated using traditional sensory evaluation techniques, following the methodology proposed by Mardiana et al. [16]. Reviewers classified the aroma descriptors for each sample based on sensory assessment results, identifying 5 key quality descriptors that represent the aroma characteristics of the 4 samples: floral, fruity, herbal, aged, and woody. The intensity of the characteristic aroma for each sample was assessed on a scale from 0 to 6, where 0 represents no sensation, 1 indicates very weak, 2 denotes weak, 3 signifies slightly weaker, 4 suggests slightly stronger, 5 indicates strong, and 6 represents very strong. Three sensory assessments were conducted, and the intensity values for the characteristic aroma attributes were averaged to derive the final QDA results.

#### 2.3.2. Pretreatment of Tea Samples

The test samples were ground into a fine powder and screened using a 40-mesh sieve, with approximately 10 g of tea powder prepared for each sample. The prepared samples were then placed in aluminum foil bags and stored in a refrigerator at 8 °C for GC-MS analysis [17].

#### 2.3.3. Headspace Solid-Phase Microextraction of Volatiles

HS-SPME extraction instructions: A mass of 0.5 g of crushed tea sample was weighted and placed in a 20 mL headspace vial. A volume of 20 µL of Ethyl decanoate (purity 98%) working solution was added to the vial, followed by the use of a 5 mL pipette to add boiling water. Then, the vial was covered, shaken well, and equilibrated in an 80 °C oven for 10 min. Then, the pre-conditioned SPME handle was inserted and the extraction head was extended into the oven at 80 °C for 50–60 min. The headspace vial and SPME handle were removed from the oven. The extraction head was withdrawn and the handle was pulled out and manually injected at the fastest speed for GC-MS analysis [18,19].

### 2.4. Conditions for GC-MS Analysis

The chromatographic instructions are as follows: A DB-5MS capillary column (30 m, 0.25 mm × 0.25 μm, Agilent J&W Scientific, Folsom, CA, USA) was used, with high-purity helium as the carrier gas at a flow rate of 1 mL/min. The inlet temperature was set to 250 °C with non-split injection, and a solvent delay of 5 min was applied. The temperature program was as follows: First, the temperature was held at 60 °C for 2 min and then heated to 220 °C at a rate of 3 °C/min and held for 10 to 20 min; finally, it was increased to 280 °C. Mass spectrometry conditions were as follows: electron bombardment ion source (EI) was utilized, ion source temperature was set as 230 °C, interface temperature was 280 °C, electron energy of 70 eV was selected, scanning mode in full scan (SCAN), and mass scanning range of *m*/*z* 40–400 was used.

### 2.5. OAV Calculation for Volatiles

The ratio of mass concentration to the odor threshold (Overflow Threshold, OT) was calculated to obtain the OAV value for each compound in the sample. The OT value was obtained from the odor threshold [20] of each volatile compound dissolved in water. The compound with OAV >1 was identified as the key aroma-active substance [21] in the sample, making a significant contribution to its characteristic aroma.

OAV value = C/T

In the formula, C represents the content of aroma components (μg/kg); T is the sensory threshold (μg/kg) of the substance in a water-soluble environment.

When the OAV is greater than 1, these ingredients are classified as key flavor components, which have a direct impact on overall flavor. If the OAV falls between 0.1 and 1, these ingredients are considered modifying flavor components; those with an OAV less than 0.1 are classified as potential flavor components with no significant impact on overall flavor. Generally, larger OAV values indicate a greater contribution of the aroma component to the overall flavor [22].

### 2.6. Data Processing

The GC-MS data were preprocessed using Microsoft Excel 2010. One-way ANOVA was performed using SPSS 20.0. The data were then imported into SIMCA 14.1 for principal component analysis (PCA) and orthogonal partial least squares-discriminant analysis (OPLS-DA). The heat map cluster analysis was created using the Maivey Cloud platform (https://www.chiplot.online, accessed on 29 October 2024). Plots were drawn using the Graphpad Prism 9.0 software.

## 3. Results

### 3.1. Results of Quantitative Descriptive Analysis

**Figure 2:**
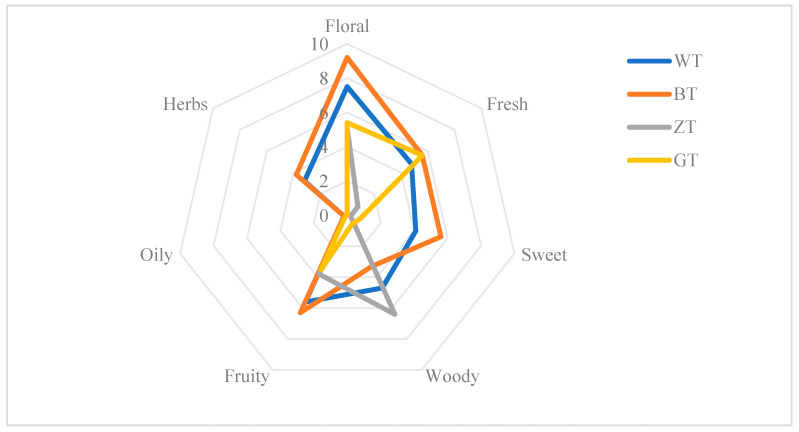
Aroma profile of different Dixu teas by QDA (WT: white tea; BT: black tea; ZT: zhutong tea; GT: green tea).

In QDA (Quantitative Descriptive Analysis), the total intensity values of aroma characteristics can provide insight into the advantages and disadvantages of aroma profiles of a sample set. The standard deviation of aroma intensity values is negatively correlated with tea flavor stability, which can be used to assess the stability [23]. The QDA results indicated that all four samples exhibited fragrance, although the intensity varied (Figure 2). The BT (black tea) group exhibited a strong and sweet fragrance compared to the WT (white tea), ZT (zhutong tea), and GT (green tea) groups. The WT group also exhibited fragrance, while the ZT group displayed a strong woody aroma, and the GT group presented a distinctive aroma. The characteristic aroma intensity values of the four sample groups ranked as follows: BT (34) > WT (30) > ZT (16.1) > GT (16). The standard deviations for the aroma intensity values were as follows: “sweet” (2.25) > “wood” (2.12) > “fragrance” (1.98) > “herbal” (1.76) > “flower” (1.67). This indicates that the “sweet” aroma exhibits low stability, whereas the “flower” aroma demonstrates the highest stability. Overall, the BT group outperformed the other three groups in terms of the “floral,” “herbal,” and “fruity” aroma attributes. These findings suggest that black tea from the fermentation industry is more conducive to producing a diverse range of aromas.

### 3.2. Analysis of Volatile Aroma Components

**Figure 3:**
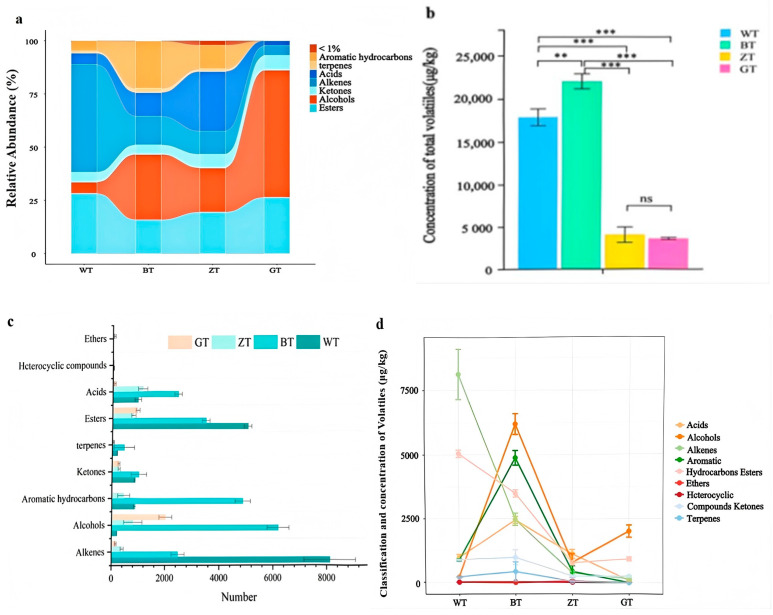
(**a**) The proportion of volatile components in four types of tea samples. (**b**) Histogram of total concentration of volatile compounds in four samples. Significance levels are represented as ** *p* < 0.01, *** *p* < 0.001, ns: not significant. (**c**) Volatile aroma types of four sets of samples. (**d**) Four different types of aroma content line graphs.

As shown in Table S1, a total of 67 aroma compounds were identified across four sets of aroma samples, including 7 alcohols, 6 ketones, 10 aldehydes, 15 esters, 7 aromatic hydrocarbons, 6 alkenes, 3 terpenes, and 10 acids. The results indicate the presence of different aroma composition types among the four DiXu tea samples processed in various methods. Further analysis of these samples is presented in Figure 3a. Consequently, both the total quantity and percentage of different aroma compounds varied significantly. In the WT group, the aldehyde and ester concentrations were higher, measuring 8118.27 µg/kg and 5046.87 µg/kg, which accounted for 45.46% and 28.26% of the total aroma compounds, respectively. In the BT group, the concentrations of aldehydes, aromatic hydrocarbons, acids, and esters were 2477.38 µg/kg, 4877.24 µg/kg, 2454.63 µg/kg, and 3488.76 µg/kg, respectively, accounting for 11.27%, 22.18%, 11.16%, and 15.86% of the total aroma compounds. In the ZT group, the concentrations were 1128.61 µg/kg and 781.83 µg/kg, which accounted for 28.12% and 19.48% of the total aroma compounds, respectively. This is consistent with the study by Liu [10]. The GT group exhibited the highest levels of alcohols, measuring 2015.39 µg/kg, which accounted for 56.37% of the total aroma compounds. The total quantities and classifications of the aroma components in the different tea samples varied significantly, as shown in Figure 3b,c. The total amounts of aroma components in the four groups were as follows: BT (21,991.51 ± 41.91 µg/kg) > WT (17,858.31 ± 62.25 µg/kg) > ZT (4014.15 ± 19.32 µg/kg) > GT (3564.49 ± 159.60 µg/kg). The total quantities of different aroma components varied significantly among the four groups (Figure 3d). The levels of aldehydes and esters in the WT group were significantly higher than those in the other groups, while the olefin levels in the BT group were significantly higher than those in the other three groups.

### 3.3. Multivariate Statistical Analysis of Volatile Aroma Components

**Figure 4:**
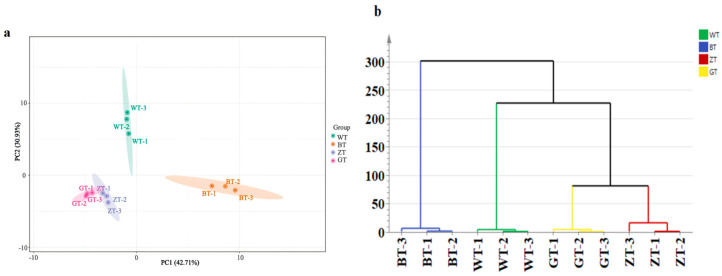

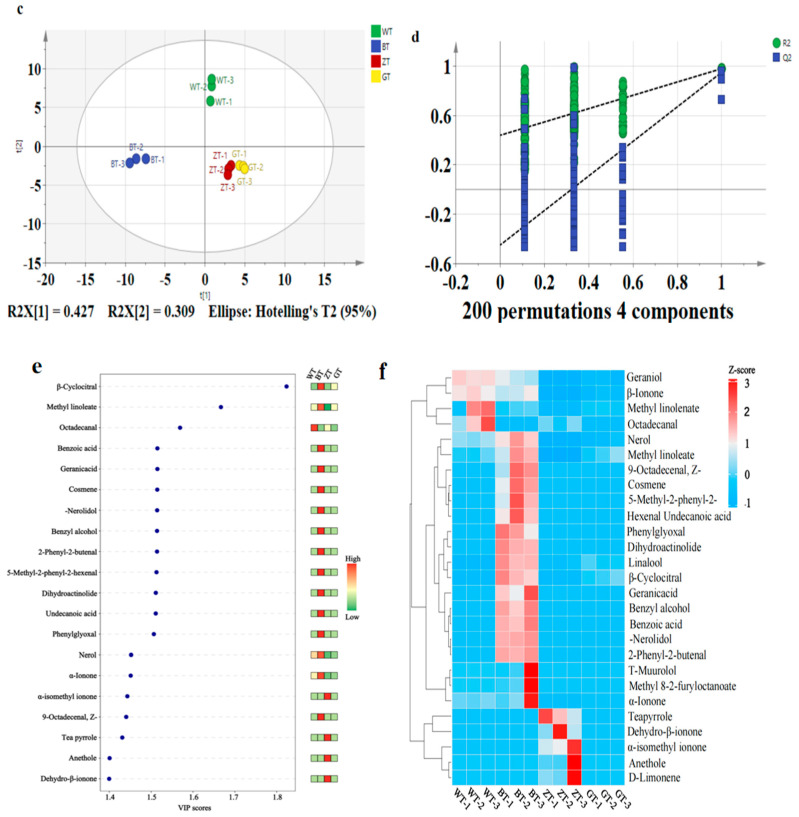
(**a**) Scatter plot of PCA model scores of volatile compounds of tea samples. (**b**) Hierarchical cluster analysis (HCA) plot. (**c**) OPLS–DA model score of volatile compounds of tea samples. (**d**) OPLS-DA permutation test chart. (**e**) Differential compounds’ VIP value plot WT vs. BT vs. ZT vs. GT. (**f**) Cluster analysis heatmap.

Principal component analysis (PCA) is an unsupervised method that maximizes data variability, reduces high-dimensional data to lower dimensions, and ensures orthogonality between dimensions. The reliability of the PCA model improves when the cumulative ratio of principal components approaches 1. After identifying 67 aroma components, we conducted a PCA for dimensionality reduction and performed a hierarchical cluster analysis (HCA analysis, Figure 4a,b). The PCA results revealed clear differences among the four sample groups, with the PC1 variance contributing 42.71%, PC2 variance contributing 30.93%, and a cumulative variance of 73.64%, which indicates the reliability of the PCA model. The HCA further confirmed the distinct clustering of the four sample groups, highlighting significant differences in the guiding aromas of the various processed teas. Specifically, the WT group significantly differed from the GT and BT, as well as the ZT groups, indicating variations in the aroma composition.

To further investigate the differential aroma components among the four sample sets, an OPLS-DA analysis of the 67 aroma components (Figure 4c) was conducted. It effectively identified four tea samples with different processing methods, using 67 aroma components as dependent variables and different tea classes as independent variables. The screening criteria for differential aroma components were based on VIP > 1 and *p* < 0.05, as detailed in the OPLS-DA model. The model’s prediction index (Q^2^) was 0.931, the fitting index for independent variables (R^2^x) was 0.882, and the fitting index for dependent variables (R^2^y) was 0.981. The model fits were acceptable when both R^2^ and Q^2^ exceeded 0.5. After conducting 200 substitution tests, as shown in Figure 3d, the intersection of the Q^2^ regression line and the vertical axis was less than 0. All of these results indicate effective model verification, demonstrating high stability, good predictive ability, and no overfitting. The heatmap illustrates the differences in the aroma compound expression levels among the four types of processed tea samples (WT, ZT, GT, and BT). The concentration distribution of specific compounds is visually represented by the color intensity, with red indicating high concentration and blue indicating low concentration. It is clearly observable in the figure that the WT group exhibits higher concentrations of aroma compounds such as Linalool, β-Cyclocitral, and Geraniol, while the GT and BT groups show more significant levels of compounds like *α*-Ionone and *β*-Ionone. These significant intergroup differences reflect the notable impact of different processing methods on the composition of tea aroma compounds.

### 3.4. OAV Analysis of Differential Aroma Compounds

The GC-MS analysis successfully characterized and quantified the volatile aroma components. However, it could not specifically identify the characteristic aroma compounds that contributed to the aroma profiles of the four sample groups. To mitigate this limitation, the OAV (odor activity value) of the volatiles was employed to identify the major volatiles present in the four samples subjected to different fermentation methods. A total of 43 aromatic compounds were identified by calculating the OAV values for the potentially labeled aromatic compounds (Table S2). These compounds were responsible for a variety of sensory aromas, and included (E)-beta-farnesene, α-Ionone [24], β-Ionone [25], and benzaldehyde [26]. Furthermore, the analysis revealed additional aromatic compounds with distinct characteristics, such as acetophenone [27], which is associated with an almond aroma, and γ-nonoltone [28], which is characterized by a coconut aroma

**Figure 5:**
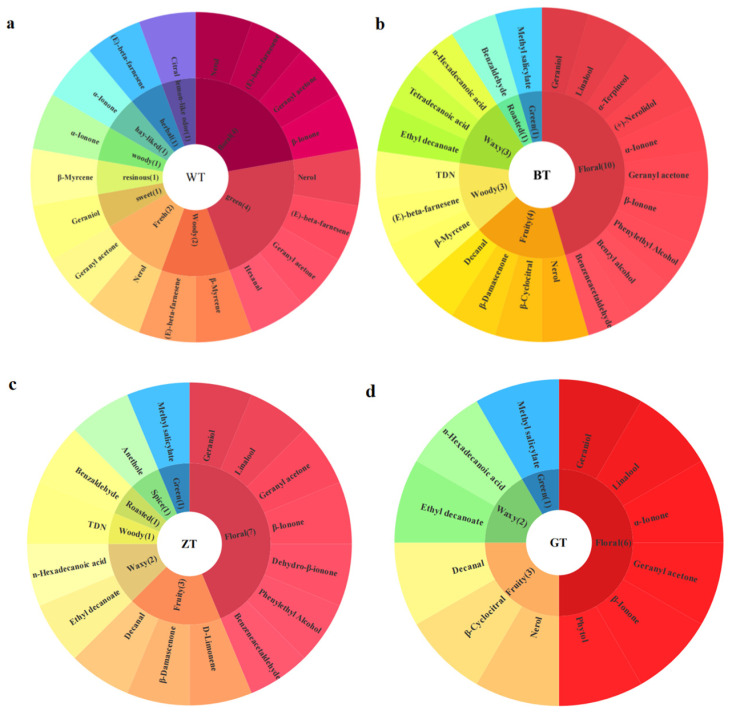

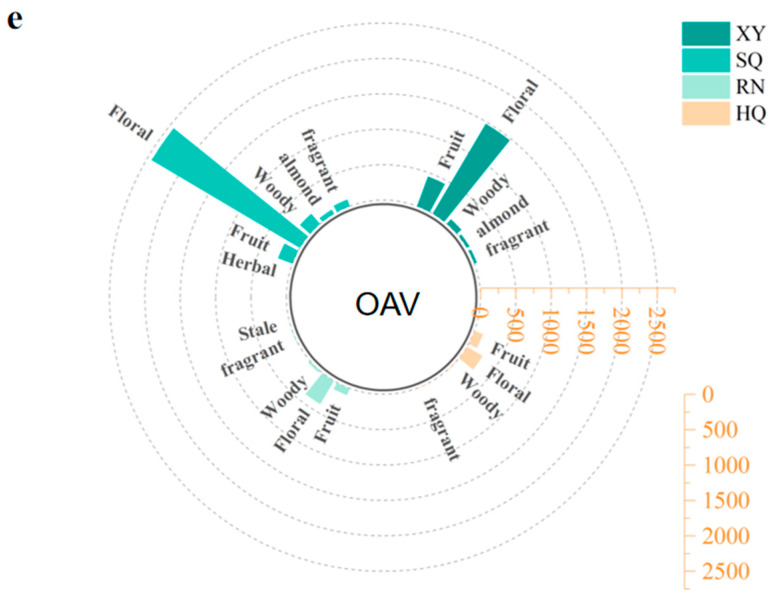
Flavor wheel of key volatile compounds in the 4 types of Dixu tea samples. (**a**) WT group; (**b**) BT group; (**c**) ZT group; (**d**) GT group; (**e**) cumulative quantitative bar chart of OAV.

Four different groups of samples were screened for key aromatic compounds with an OAV greater than 1 to create a flavor wheel. The OAV of the characteristic aroma compounds across the different aroma types in each group was further quantified (Figure 5e). In the four groups of Dixu tea samples, the cumulative OAV quantified across the different aroma types showed variability. However, the cumulative OAV for the fruit aroma characteristics was significantly higher. Aroma is a key characteristic of high-quality Dixu tea and is positively correlated with its overall quality.

**Figure 6:**
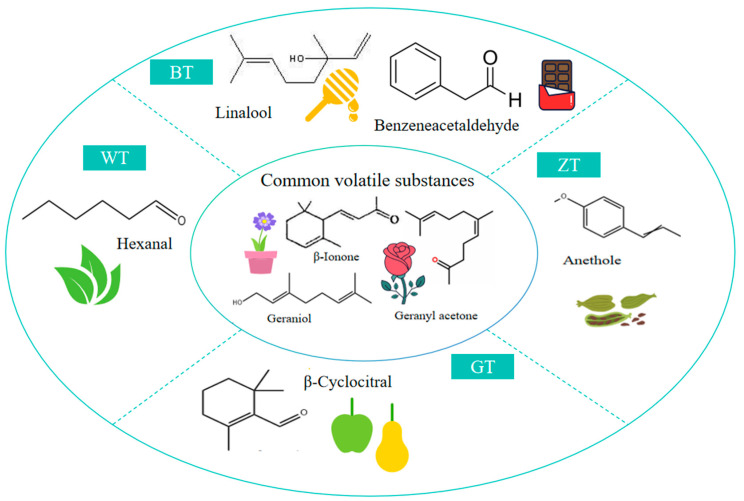
The key volatile compounds in the 4 types of samples (OAV > 10).

The aromatic intensity of a compound is directly correlated with its OAV: the higher the OAV, the more pronounced the compound’s impact on the aroma of the sample. A compound is considered to make an important contribution to the overall aroma of the tea when its OAV exceeds 10 [29]. Using OAV >10 as the screening criterion, the key characteristic aroma compounds were further selected [30], as shown in Figure 6. The analysis revealed that the common aromas of the four types of tea produced in Dixu, Guangnan, are β-ionone, geranyl acetone, and geraniol. These volatile compounds were detected in all four types of tea and are characterized by floral aromas, which is consistent with the results of the sensory evaluation. Therefore, it was concluded that a floral aroma is the common characteristic of the four types of tea from Guangnan Dixu. According to Table S2 and Figure 6, in addition to the floral aroma, each tea type also exhibits its own characteristic aromas. For example, hexanal in the WT group imparts a fresh and fruity aroma, making a significant contribution to the fruity characteristic of this group. In the BT group, the characteristic compounds are linalool, which provides a sweet aroma, and benzeneacetaldehyde, which contributes a fresh and chocolate-like fragrance. In contrast, the ZT group contains anethole, which offers a spicy and woody aroma. All these conclusions are consistent with the sensory evaluation results. Notably, the aroma profile of the BT group demonstrates greater diversity compared to the other three groups. This result may be due to the fermentation process, as fermentation increases the abundance of aroma compounds.

## 4. Discussion

Aroma, as one of the most important factors affecting tea quality, is also an important factor in consumers’ sensory experience, influenced by various factors such as the tea tree variety, processing technology, growth environment, and fresh leaf quality. The adaptability of different tea tree varieties varies [31]. Those with higher phenol ammonia ratios are suitable for producing black tea, while those with lower ratios are suitable for producing green tea [32]. The vastly different proportions of internal substances determine the differences in aroma among the different tea tree varieties. The vastly different proportions of internal substances determine the differences in aroma among the different tea tree varieties. The method for detecting aroma usually uses distillation extraction (SDE) to extract volatile components from tea, and then separates and identifies them using gas chromatography-mass spectrometry (GC-MS) [33]. This study investigated the volatile compounds of different types of tea produced in Guangnan Diwei tea using QDA, HS-SPME-GC/MS, and multivariate statistical methods, combined with a sensory analysis.

Quantitative Descriptive Analysis (QDA), as a commonly used method for evaluating the characteristic properties of samples, can more accurately and intuitively describe the sensory attributes of samples, making it a comprehensive sensory method that can quantify sensory evaluation results. This method has been widely used for the sensory evaluation of various tea samples [15]. In order to conduct a more comprehensive and scientific analysis of the aroma of the samples, the QDA method was used to further evaluate the specific aroma characteristics of the samples based on traditional sensory evaluation [16]. In QDA (Quantitative Descriptive Analysis), the total intensity values of aroma characteristics can provide insight into the advantages and disadvantages of the aroma profiles of a sample set. The QDA results indicated that all four samples exhibited fragrance, although the intensity varied (Figure 1). The BT (black tea) group exhibited a strong and sweet fragrance compared to the WT (white tea), ZT (zhutong tea), and GT (green tea) groups. The WT group also exhibited fragrance, while the ZT group displayed a strong woody aroma, and the GT group presented a distinctive aroma.

In recent years, based on headspace solid-phase microextraction gas chromatography mass spectrometry, analytical techniques such as chromatography mass spectrometry (HS-SPME-GC MS) have been combined with principal component analysis (PCA), partial least squares discriminant analysis (orthogonal partial least squares-discriminant analysis, OPLS-DA), and hierarchical cluster analysis (HCA). By using statistical analysis methods, the effects of different colors or grades [34], different storage times [35], different origins [36], and different aroma types [37] of tea samples can differentiated and used to screen for key differential metabolites formed as a result of them. This study conducted an in-depth analysis of the aroma components from different tea samples (WT, BT, ZT, and GT) and identified 67 aromatic compounds through HS-SPME-GC/MS technology. Among them, the BT tea sample exhibited the highest aroma intensity with a prominent fruity aroma, while WT and GT stood out for their floral characteristics, and ZT showed distinct woody notes due to its unique processing methods. The OAV analysis revealed that sesquiterpenes (e.g., β-ionone and linalool oxides) and ester compounds (e.g., octanoate and methyl salicylate) significantly contributed to the overall flavor profile of the different tea samples. The multivariate analysis demonstrated that the processing methods had a significant impact on the tea sample aromas. The PCA explained 73.64% of the total variance. In addition, 17 key differential aromatic compounds (VIP > 1, *p* < 0.05), such as limonene, trans-2-hexenal, and linalool, were identified via OPLS-DA, effectively distinguishing the tea types and processing methods.

Combining sensory evaluation and chemical analysis, BT’s “fruity” and “floral” notes were highly prominent, while WT and GT had more floral contributions from aromatic compounds. ZT’s woody aroma correlated with its unique processing technique. Moreover, the interplay between the threshold values and OAVs indicated that certain components might act as modifiers or latent contributors to the aroma, suggesting that their synergistic effects and perceptual interactions warrant further study.

## 5. Conclusions

This study analyzed the aromatic volatile substances of WT, BT, ZT, and GT produced from Guangnan Diwei tea, and detected differences in volatile substances among the different types of tea. Subsequent research could combine electronic noses, electronic tongues, GC-O-MS, aroma omission and recombination experiments, S-curve fitting, and other methods to compare Guangnan Diwei tea produced in different seasons and using different processing techniques, and further analyze the contribution of different aroma compounds to the overall aroma and the perceptual interaction between compounds, in order to provide a reference for improving the aroma of Guangnan Dixu tea.

## Data Availability

Data will be made available upon request from the corresponding author. The data are not publicly available due to privacy.

## Supplementary Materials

The following supporting information can be downloaded at https://www.mdpi.com/article/10.3390/metabo15040257/s1: Table S1: Volatile compounds of different tea types in Guangnan Dixu tea, Table S2: Volatile organic compounds (OAV) of different tea types in Guangnan Dixu tea. Figure S1. The TIC (Total Ion Chromatogram) of the white tea (WT) sample. Figure S2. The TIC (Total Ion Chromatogram) of the black tea (BT) sample. Figure S3. The TIC (Total Ion Chromatogram) of the zhutong tea (ZT) sample. Figure S4. The TIC (Total Ion Chromatogram) of the green tea (GT) sample. Table S3 Detailed information of the volatile compounds in white tea (WT). Table S4 Detailed information of the volatile compounds in black tea (BT). Table S5 Detailed information of the volatile compounds in zhutong tea (ZT). Table S6 Detailed information of the volatile compounds in green tea (GT).
