# Supplementary material for: Aromatic Volatile Substances in Different Types of Guangnan Dixu Tea Based on HS-SPME-GC-MS Odor Activity Value
Source: Metabolites. 2025 Apr 9;15(4):257. doi: 10.3390/metabo15040257 (PMC12029467; doi:10.3390/metabo15040257)
Supplement: Supplementary file 1 [file metabolites-15-00257-s001.zip › metabolites-3504691-supplementary.pdf]

Supporting Information

Research on Aromatic Volatile Substances of Different Tea Types in Guangnan Dixu Tea Based on HS-SPME-GC-MS OAV

Ying Feng [1], Di Tian[2], Chaoliang Wang[1],Yong Huang[1] ,Yang Luo[1],Xiuqiong Zhang [3*], Lei Li [1*]

[1] College of Sanqi Medical, Wenshan College Wenshan 663099, China

[2] College of Food Science and Technology, Yunnan agricultural university Kunming , 650051 ,China

[3] College of Chemistry and Chemical Engineering, Yunnan Normal University, Kunming 650500. China

*** Corresponding Authors:**

Dr. Lei Li, College of Sanqi Medicine, Wenshan University, Research Interests: Food Processing and Safety186, Wenshan 663000, China. Tel: 18608769508. E-mail: fy1085040603@outlook.com.

Dr. Xiuqiong Zhang, College of Chemistry and Chemical Engineering, Yunnan Normal University, Kunming 650500, China. Tel: 0871-65941087/ Fax: 0871-65941088. E-mail: zhangxiuqiong@ynnu.edu.cn.

Table S1 Volatile compounds of different tea types in Guangnan Dixu tea.

Serial Number	Classification of substances	Compound name	CAS	potency (μg/L)			
				WT	BT	ZT	GT
1	Alkenes	*β*-Myrcene	123-35-3	440.08±23.22[a]	303.34±78.03[b]	--	6.68±3.54[c]
2	Alkenes	1,3,8-p-Menthatriene	018368-95-1	208.07±50.13[a]	--	--	--
3	Alkenes	Neo-alloocimene	7216-56-0	268.54±15.66[a]	--	--	--
4	Alcohols	Nerol	106-25-2	113.27±10.60[b]	202.68±30.15[a]	--	26.24±14.11[c]
5	Alkenes	Geraniol	106-24-1	731.27±21.84[a]	552.21±67.77[b]	31.20±1.69[d]	112.53±29.92[c]
6	Alkenes	Citral	5392-40-5	155.38±12.25[a]	--	--	--
7	Alcohols	Linalool	78-70-6	--	5259.27±504.24[a]	412.04±195.54[c]	1411.63±257.28[b]
8	Alkenes	Cosmene	460-01-5	--	157.4488±49.1084[a]	--	--
9	Alcohols	*α*-Terpineol	98-55-5	--	208.90±134.57[a]	88.96±15.58[ab]	38.46±7.05[b]
10	Alkenes	D-Limonene	5989-27-5	--	--	38.55±42.27[a]	--
11	Alcohols	4-Terpinenol	562-74-3	--	--	--	94.37±8.47[a]
12	terpenes	*α*-Cadinol	481-34-5	--	--	--	40.76±25.52[a]
13	Alcohols	*α*-Cedrene	469-61-4	62.13±3.11[a]	90.62±36.50[a]	51.04±18.68[a]	--
14	terpenes	(E)-*β*-farnesene	77129-48-7	94.01±4.12[a]	111.56±21.09[a]	--	--
15	terpenes	T-Muurolol	19912-62-0	34.47±5.77[a]	244.66±353.23[a]	--	--
16	Alcohols	(+)-Nerolidol	1285-43-6	--	342.9433±19.7775[a]	--	--
17	Ketones	*α*-Ionone	127-41-3	113.86±11.66[ab]	264.82±259.47[a]	--	26.67±3.45[ab]
18	Ketones	Geranyl acetone	3796-70-1	117.47±7.87[a]	96.58±20.53[a]	54.66±8.27[c]	29.55±8.14[d]
19	Ketones	*β*-Ionone	14901-07-6	622.19±33.37[a]	539.31±54.73[b]	103.80±11.37[d]	194.35±30.26[c]
20	Alkenes	*β*-Cyclocitral	432-25-7	--	223.88±34.91[a]	--	68.24±15.23[b]
21	Hcterocyclic compou--s	TDN	30364-38-6	--	34.62±6.78[a]	17.57±3.41[b]	--
22	Acids	Geranic acid	4698-08-2	--	145.57±54.18[a]	--	--
23	Ketones	*β*-Damascenone	23726-93-4	--	103.59±9.00[a]	23.36±9.07[b]	--
24	Ketones	α-isomethyl ionone	6901-97-9	--	--	44.35±25.20[a]	--
25	Ketones	Dehydro-*β*-ionone	1203-08-3	--	--	36.13±31.56[a]	--
26	Alkenes	Benzaldehyde	100-52-7	1581.56±596.03[a]	2098.75±200.98[a]	287.85±35.82[b]	--
27	Aromatic hydrocarbons	Phenylethyl Alcohol	60-12-8	852.12±33.92[b]	1760.66±324.12[a]	232.82±86.36[c]	--
28	Aromatic hydrocarbons	Benzyl alcohol	100-51-6	--	555.13±87.84[a]	--	--

29	Aromatic hydrocarbons	Benzeneacetaldehyde	122-78-1	--	1592.21±301.62[a]	33.52±20.65[b]	--
30		Phenylglyoxal	1074-12-0	--	306.70±79.42[a]	--	--
31	Acids	Benzoic acid	65-85-0	--	119.59±17.40[a]	--	--
32	Aromatic hydrocarbons	2-Phenyl-2-butenal	4411-89-6	--	375.53±25.11[a]	--	--
33	Aromatic hydrocarbons	5-Methyl-2-phenyl-2-hexenal	21834-92-4	--	167.42±67.15[a]	--	--
34	Aromatic hydrocarbons	Anethole	104-46-1	--	--	177.57±239.25[a]	--
35		Methyl salicylate	119-36-8	3415.68±258.02[a]	2182.20±222.80[a]	280.02±92.17[b]	282.57±56.21[b]
36	Esters	Ethyl decanoate	110-38-3	394.70±3.63[a]	345.08±121.55[a]	396.79±6.60[a]	287.53±94.38[a]
37	Esters	trans-Nerolidyl formate	1429-39-7	276.89±23.51[a]	--	--	--
38	Esters	Hexyl benzoate	6789-88-4	19.89±1.50[a]	--	--	--
39	Esters	Methyl 8-(2-furyl)octanoate	38199-50-7	55.59±2.01[a]	346.59±447.33[a]	--	--
40	Esters	Methyl tetradecanoate	124-10-7	40.41±0.85[a]	50.30±12.37[a]	--	--
41	Esters	Methyl hexadecanoate	112-39-0	385.62±121.78[a]	201.73±24.37[bc]	71.90±3.94[c]	231.08±101.37[b]
42	Esters	Methyl linoleate	2462-85-3	23.46±2.73[b]	53.18±12.61[a]	--	24.91±6.87[b]
43	Esters	Methyl linolenate	301-00-8	232.51±171.47[a]	81.30±15.31[ab]	--	55.66±8.64[b]
44	Esters	Methyl isostearate	5129-61-3	60.10±69.09[a]	--	--	--
45	Esters	Butyl hexadecanoate	111-06-8	33.68±3.07[ab]	18.36±4.11[b]	23.50±11.60[ab]	36.92±12.02[a]
46	Esters	Butyl octadecanoate	123-95-5	38.54±32.75[a]	--	--	--
47	Esters	Methyl stearate	112-61-8	--	--	9.63±3.50[b]	23.56±2.73[a]
48	Acids	Nonanoic acid	112-05-0	152.96±11.21[c]	818.29±77.22[a]	343.00±52.49[b]	--
49	Acids	n-Decanoic acid	334-48-5	150.02±76.75[ab]	229.16±32.55[a]	83.38±17.99[b]	--
50	Acids	Dodecanoic acid	143-07-7	261.29±7.18[b]	710.14±153.05[a]	302.29±9.90[b]	--
51	Acids	Tetradecanoic acid	544-63-8	69.23±29.21[ab]	93.51±26.73[a]	49.60±7.62[b]	--
52	Acids	Pentadecanoic acid	1002-84-2	122.09±91.71[a]	--	--	--
53	Acids	n-Hexadecanoic acid	57-10-3	167.99±51.69[ab]	217.70±19.41[a]	116.38±15.43[bc]	83.95±27.51[c]
54	Acids	Octadecanoic acid	57-11-4	25.63±21.345[a]	--	--	--
55	Acids	Octanoic acid	124-07-2	--	177.02±43.915[a]	233.97±116.30[a]	--
56	Acids	U--ecanoic acid	112-37-8	--	63.24±20.44a	--	--
57	Alkenes	Hexanal	66-25-1	2748.58±660.48[a]	--	--	--
58	Alkenes	(E)-2-Hexenal	6728-26-3	3532.17±258.97[a]	--	--	--
59	Alkenes	Octadecanal	638-66-4	100.55±50.72[a]	--	32.75±14.97[b]	--
60	Alkenes	Decanal	112-31-2	--	123.05±39.97[a]	42.36±12.93[b]	41.42±19.62[b]
61	Alkenes	9-Octadecenal, (Z)-	2423-10-1	--	5.47±2.45[a]	--	--
62	Alkenes	E-15-Heptadecenal	1130-97-9	--	26.22±8.61[a]	29.60±19.80[a]	--
63	Alkenes	Tetradecanal	124-25-4	--	--	--	43.48±21.54[a]

64	Esters	*γ*-nonalactone	104-61-0	69.83±18.13[a]	--	--	--
65	Esters	Dihydroactinolide	15356-74-8	--	210.03±22.11[a]	--	--
66	Ethers	Tea pyrrole	2167-14-8	--	--	67.22±30.48[a]	--
67	Alcohols	Phytol	150-86-7	86.49±10.32[c]	180.95±26.04[bc]	298.34±134.20[ab]	403.93±137.52[a]

Note: - -This means that it is not detected: a> 0.05, * b < 0.05, **: c < 0.01, ***: d < 0.001

Table S2 Volatile organic compounds (OAV) of different tea types in Guangnan Dixu tea.

Serial Number	Volatile compound name	threshold	Odor quality	OAV			
				WT	BT	ZT	GT
1	β-Myrcene	15.00	Woody, resinous, musty	29.34	20.22	0.00	0.45
2	1,3,8-p-Menthatriene	15.00	turpentine camphor herbal woody	13.87	0.00	0.00	0.00
3	Nerol	49.00	Fresh, citrus, floral, green, lemon-like	2.31	4.14	0.00	0.54
4	Geraniol	7.50	Rose-like, sweet, honey-like	97.50	73.63	4.16	15.00
5	Citral	30.00	lemon-like odor	5.18	0.00	0.00	0.00
6	Linalool	0.22	Floral, sweet, grape-like, woody	0.00	23905.77	1872.89	6416.52
8	D-Limonene	10.00	Fruity, lemon-like	0.00	0.00	3.86	0.00
11	(E)-β-farnesene	87.00	Woody, green, floral, herbal	1.08	1.28	0.00	0.00
12	(+)-Nerolidol	0.25	Floral, green, citrus, woody, waxy	0.00	1371.77	0.00	0.00
13	α-Ionone	0.40	woody, hay-liked	284.66	662.06	0.00	66.66
14	Geranyl acetone	0.06	Fresh, rose-like, floral, green, fruity	1957.82	1609.74	911.00	492.46
15	β-Ionone	0.09	floral, woodyc	6913.21	5992.30	1153.30	2159.43
16	β-Cyclocitral	3.00	Herbal, clean, rose-like, fruity	0.00	74.63	0.00	22.75
17	TDN	2.50	Licorice-like	0.00	13.85	7.03	0.00
18	β-Damascenone	0.002	Fruity, apple-like	0.00	51792.96	11681.40	0.00
19	Dehydro-β-ionone	0.09	Violet, woody, raspberry	0.00	0.00	401.40	0.00
20	Benzaldehyde	3.00	almond-like smelle	527.20	699.58	95.95	0.00
21	Phenylethyl Alcohol	45.00	rose, floralc	18.94	39.13	5.17	0.00
22	Benzyl alcohol	100.00	Burning taste, faint aromatic	0.00	5.55	0.00	0.00
23	Benzeneacetaldehyde	4.00	clean aroma; rose-like, floral and chocolate-like smelle	0.00	398.05	8.38	0.00
26	Anethole	15.00	sweet anise licorice mimosa	0.00	0.00	11.84	0.00
27	Methyl salicylate	40.00	minty flavord, Wintergreen like	85.39	54.56	7.00	7.06
35	n-Hexadecanoic acid	10.00	Waxy, creamy, candle-like	16.80	21.77	11.64	8.39
38	Hexanal	4.50	green, grassy, fruitye	610.79	0.00	0.00	0.00
39	(E)-2-Hexenal	190.00	Green	18.59	0.00	0.00	0.00

40	Decanal	0.10	aldehyde-like, waxy, fatty and citrus-likee	0.00	1230.53	423.63	414.19
42	*γ*-nonalactone	65.00	coconut, waxy sweet butterye	1.07	0.00	0.00	0.00
43	Phytol	640.00	floral, balsam, powdery, waxy	0.14	0.28	0.47	0.63

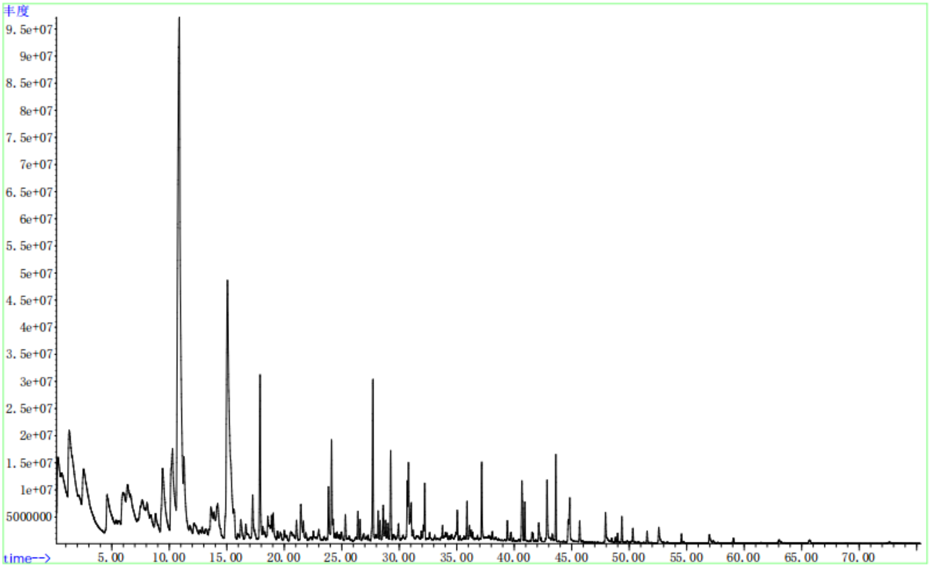

Figure S1. The TIC (Total Ion Chromatogram) of the white tea (WT) sample.

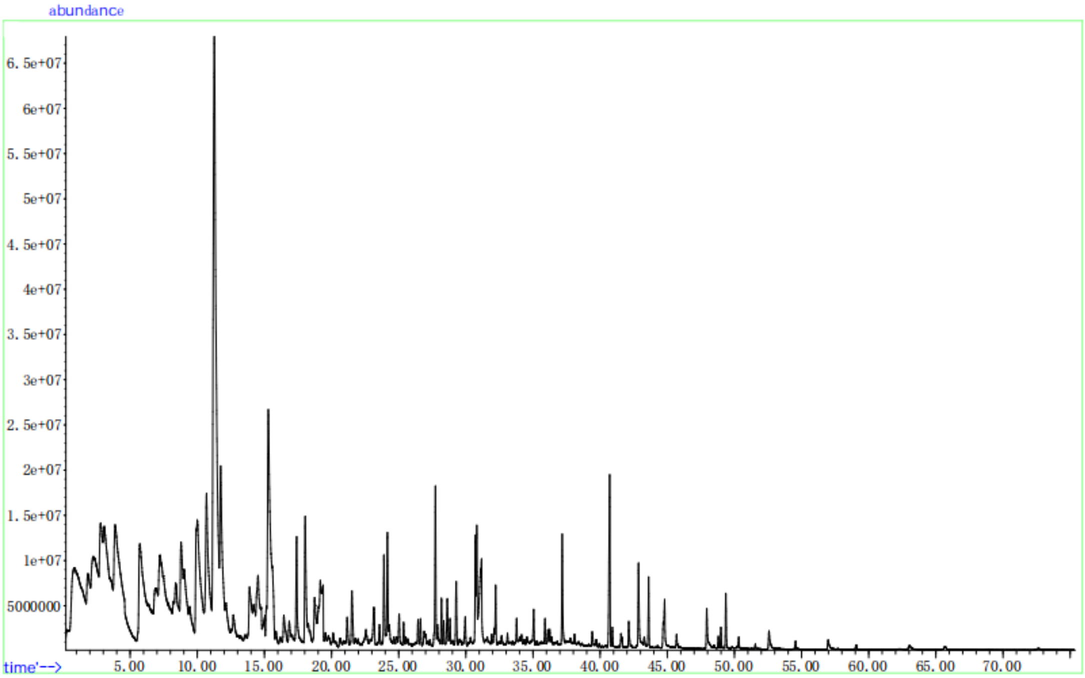

Figure S2. The TIC (Total Ion Chromatogram) of the black tea (BT) sample.

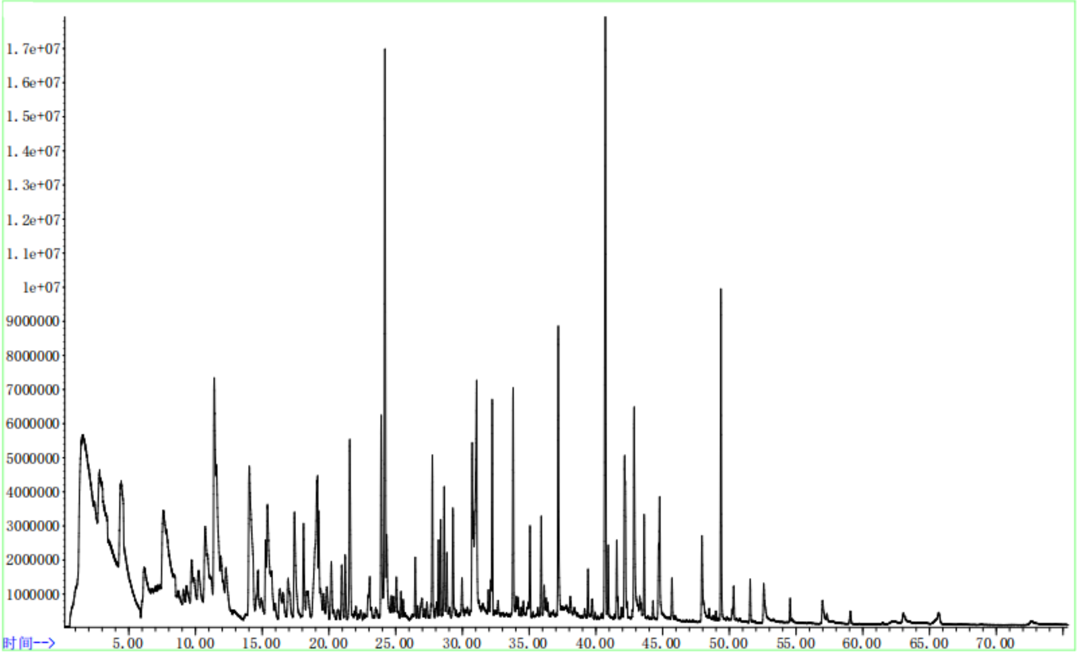

Figure S3. The TIC (Total Ion Chromatogram) of the zhutong tea (ZT) sample.

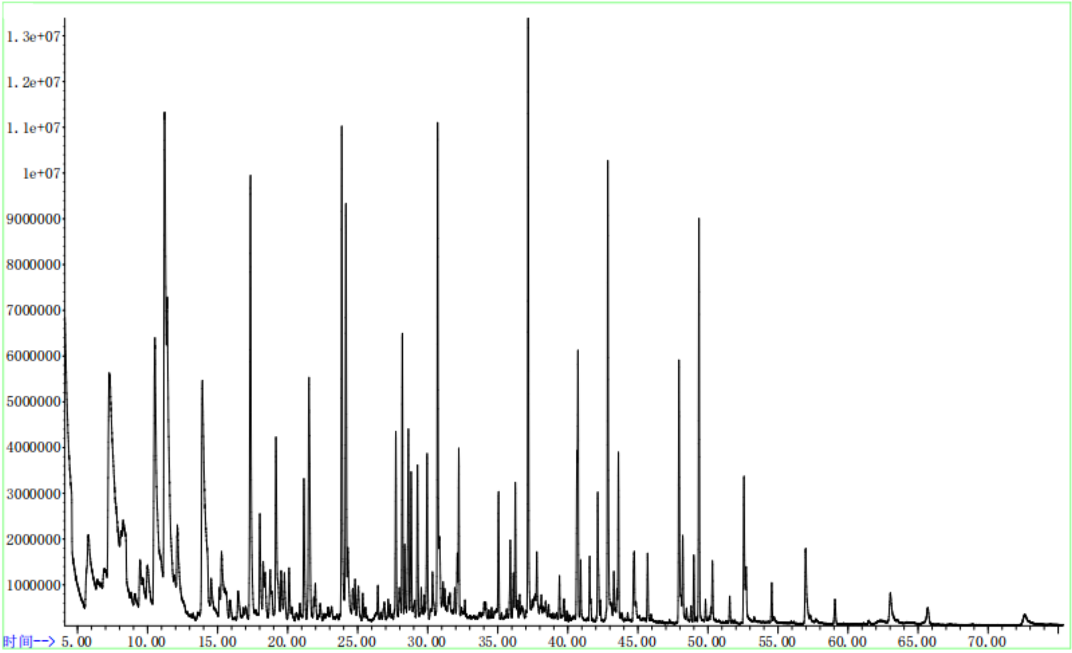

Figure S4. The TIC (Total Ion Chromatogram) of the green tea (GT) sample.

Table S3 Detailed information of the volatile compounds in white tea (WT).

Number	Time	Compound name	Molecular formula	CAS	Peak height	Peak area
1	0.32	Hexanal	C6H12O	000066-25-1	16010628	6818505802
2	1.29	(E)-2-Hexenal	C6H10O	006728-26-3	20858531	9273058730
3	4.59	Benzaldehyde	C7H6O	000100-52-7	9148548	3518310307
4	6.02	β-Myrcene	C10H16	000123-35-3	9486370	1162096053
5	11.28	Phenylethyl Alcohol	C8H8O	000060-12-8	16108555	2217104524
6	12.18	1,3,8-p-Menthatriene	C10H14	018368-95-1	3900887	456847939
7	12.29	(E,Z)-2,6-dimethyl-2,4,6-Octatriene	C10H16	007216-56-0	3448890	684578285
8	15.06	Methyl salicylate	C8H8O3	000119-36-8	48650141	9471996059
9	16.65	(Z)- 3,7-dimethyl- 2,6-Octadien-1-ol	C10H18O	000106-25-2	3694197	294016427
10	16.81	3-ethyl-4-methyl-2,5-dione-1H-Pyrrole	C7H9NO2	020189-42-8	1906179	104863525
11	17.89	Geraniol	C10H18O	000106-24-1	31079181	1949792050
12	18.57	Citral	C10H16O	005392-40-5	5087379	397839921
13	18.914	Nonanoic acid	C9H18O2	000112-05-0	5313353	386955668
14	19.04	2,6,11-trimethyl-Dodecane	C15H32	031295-56-4	5699252	457804669
15	21.06	1-iodo-Dodecane	C12H25I	004292-19-7	4385828	301757217
16	22.53	dihydro-5-pentyl-2(3H)-Furanone	C9H16O2	000104-61-0	2384828	134217920
17	22.78	3,3,4,7-tetramethyl-Benzo	C12H14O2	037740-08-2	1271960	83579092
18	23.01	n-Decanoic acid	C10H20O2	000334-48-5	2698664	281033964
19	23.84	2-(diethoxyphosphinyl)-3-(4-N,N-Dimeth ylaminophenyl)propenoic acid	C17H26NO5P	066564-08-7	10595806	637807000
20	24.11	Decanoic acid,	C12H24O2	000110-38-3	19217420	1027831195
21	24.24	Tetradecane	C14H30	000629-59-4	4628537	273412028
22	24.59	2,3,4,7,8,8a-hexahydro-3,6,8,8-tetrameth yl-3R-1H-7-Methanoazulene,	C15H24	000469-61-4	2243774	159904670
23	25.31	α-Ionone	C13H20O	000127-41-3	5432284	292135027
24	26.40	(E)-6,10-dimethyl-5,9-Undecadien-2-one,	C13H22O	003796-70-1	6049042	303658002
25	26.59	7,11-dimethyl-3-methylene-1,6,10-Dodec atriene	C15H24	077129-48-7	4520118	250663757

Number	Time	Compound name	Molecular formula	CAS	Peak height	Peak area
26	27.16	2,5-dien-1-one-2,6-Di-tert-butyl-4-hydroxy-4-methylcyclohexa	C15H24O2	010396-80-2	1387374	99408417
27	27.29	Cyclododecane	C12H24	000294-62-2	1660917	105509259
28	27.71	4-(2,6,6-trimethyl-1-cyclohexen-1-yl)-3-Buten-2-one	C13H20O	014901-07-6	30192002	1589581728
29	28.03	1-Pentadecene	C15H30	013360-61-7	1683548	81226397
30	28.16	1-iodo-Docosane	C22H45I	1000406-31-9	6133281	288784473
31	28.33	Pentadecane	C15H32	000629-62-9	4366533	220477279
32	28.81	2,4-Di-tert-butylphenol	C14H22O	000096-76-4	4403849	264714484
33	29.77	Heneicosane	C21H44	000629-94-7	1090441	22855648
34	30.33	Heptacosane	C27H56	000593-49-7	1643937	160033407
35	30.80	trans-Nerolidyl formate	C16H26O2	1000429-39-7	15078434	701683103
36	31.04	Dodecanoic acid	C12H24O2	000143-07-7	7622317	680136576
37	31.29	hexyl ester-Benzoic acid	C13H18O2	006789-88-4	1257899	48032955
38	31.59	1-Bromodocosane	C22H45Br	006938-66-5	1528598	213906799
39	31.77	*β*-Bisabolenol	C15H24O	147126-90-7	965237	29829104
40	31.92	Cetene	C16H32	000629-73-2	2417417	147232818
41	32.21	Hexadecane	C16H34	000544-76-3	11204472	495878644
42	32.65	Z-11,13-Dimethyl-11-tetradecen-1-ol acetate	C18H34O2	1000131-36-6	2137156	148887629
43	33.09	Methyl 8-(2-furyl)octanoate	C13H20O3	038199-50-7	1654570	147593167
44	33.671	.tau.-Muurolol	C15H26O	019912-62-0	1300494	71963966
45	34.03	2,6,10,14-tetramethyl-Hexadecane	C20H42	000638-36-8	2162230	278558470
46	34.36	2,2',5,5'-tetramethyl-1,1'-Biphenyl	C16H18	003075-84-1	1529052	92385563
47	34.56	2-methyl-Hexadecane	C17H36	001560-92-5	1804855	113130676
48	34.84	2-methyl-Heptadecane	C18H38	001560-89-0	1471542	79227441
49	34.92	2-hexyl-1-Decanol	C16H34O	002425-77-6	2166222	101783433
50	35.51	Octacosyl heptafluorobutyrate	C32H57F7O2	1010351-83-6	1020149	57053274
51	35.63	(E)-5-Octadecene	C18H36	007206-21-5	1496191	104780920
52	35.895	Heptadecane	C17H36	000629-78-7	7874687	401733070
53	36.12	2,6,10,14-tetramethyl-Pentadecane	C19H40	001921-70-6	3406018	208769090
54	36.25	Hexacosane	C26H54	000630-01-3	2547047	141672022
55	36.38	Octadecanal	C18H36O	000638-66-4	2183617	134498309
56	36.56	2-Methylhexacosane	C27H56	001561-02-0	1080671	51422538
57	36.81	Methyl tetradecanoate	C15H30O2	000124-10-7	1613727	103882620

Number	Time	Compound name	Molecular formula	CAS	Peak height	Peak area
58	37.47	Eicosane	C20H42	000112-95-8	1362066	298160536
59	37.78	Pentacosane	C25H52	000629-99-2	1598129	76754596
60	38.09	Tetradecanoic acid	C14H28O2	000544-63-8	2305972	202093705
61	38.39	3-methyl-Heptadecane	C18H38	006418-44-6	1271503	100666437
62	38.63	N-(2-trifluoromethylphenyl)-oxime-Pyridine-3-carboxamide	C13H10F3N3O	288246-53-7	1002208	209784197
63	39.16	1-Nonadecene	C19H38	018435-45-5	883346	84842054
64	39.40	Nonadecane	C19H40	000629-92-5	4338665	340809120
65	39.58	Z-8-Hexadecene	C16H32	1000130-87-5	852640	33516185
66	39.94	Cyclohexadecane	C16H32	000295-65-8	1166124	66105474
67	40.67	Caffeine	C8H10N4O2	000058-08-2	11637364	743368787
68	40.92	6,10,14-trimethyl-2-Pentadecanone	C18H36O	000502-69-2	7745010	326085739
69	41.41	Pentadecanoic acid	C15H30O2	001002-84-2	743539	44161719
70	41.64	bis(2-methylpropyl) ester-1,2-Benzenedicarboxylic acid	C16H22O4	000084-69-5	2006664	80623680
71	43.62	methyl ester Hexadecanoic acid	C17H34O2	000112-39-0	16480181	671602289
72	44.69	Dibutyl phthalate	C16H22O4	000084-74-2	4500528	198252189
73	44.83	n-Hexadecanoic acid	C16H32O2	000057-10-3	8523365	537226999
75	48.477	1-Octadecene	C18H36	000112-88-9	1109099	62961984
76	48.80	methyl ester-9,12-Octadecadienoic acid	C19H34O2	002462-85-3	1284984	57367235
77	48.99	(Z,Z,Z)-methyl ester-9,12,15-Octadecatrienoic acid	C19H32O2	000301-00-8	2007075	91158034
78	49.36	Phytol	C20H40O	000150-86-7	5107458	225977601
79	49.83	16-methyl-Heptadecanoic acid	C19H38O2	005129-61-3	677096	37369172
80	50.80	Octadecanoic acid	C18H36O2	000057-11-4	494647	31217496
81	51.56	butyl ester-Hexadecanoic acid	C20H40O2	000111-06-8	2407647	95866466
82	52.58	N-[4-(1-methylethyl)benzylidene]-4-(1-pyrrolidylsulfonyl)-Benzenamine	C20H24N2O2S	314765-47-4	3068170	245827472
83	57.30	butyl ester-Octadecanoic acid	C22H44O2	000123-95-5	711605	45724784

Table S4 Detailed information of the volatile compounds in black tea(BT).

Number	Time	Compound name	Molecular formula	CAS	Peak height	Peak area
1	5.29	methoxy-phenyl-Oxime	C8H9NO2	1000222-86-6	1511857	130391965
2	5.72	Benzaldehyde	C7H6O	000100-52-7	11850149	4099444436
3	6.94	β-Myrcene	C10H16	000123-35-3	6970720	675792421
4	8.25	1,3,3-trimethyl-Tricyclo[2.2.1.0(2,6)]heptane	C10H16	000488-97-1	5499552	458273179
5	8.39	Benzyl alcohol	C7H8O	000100-51-6	7456067	1021477869
6	8.79	Benzeneacetaldehyde	C8H8O	000122-78-1	12020431	2985569276
7	9.93	Phenylglyoxal	C8H6O2	001074-12-0	13379078	681004333
8	11.27	Linalool	C10H18O	000078-70-6	67921927	10590232217
9	11.74	Phenylethyl Alcohol	C8H8O	000060-12-8	20387852	3652441505
10	12.55	1,3,5,7-octatetraene,E,E-2,6-Dimethyl	C10H14	000460-01-5	2373937	190935860
11	12.69	1-ethyl-2,5-Pyrrolidinedione	C6H9NO2	002314-78-5	3965763	549467812
12	13.04	1,2,3,4,5-pentamethyl-1,3-Cyclopentadiene	C10H16	004045-44-7	1751677	156048465
13	14.78	Benzoic acid	C7H6O2	000065-85-0	4795668	209195398
14	15.04	Octanoic acid	C8H16O2	000124-07-2	3952587	408927877
15	15.14	α-Terpineol	C10H18O	000098-55-5	4953928	235146969
16	15.29	Methyl salicylate	C8H8O3	000119-36-8	26484880	4304522698
17	15.86	Decanal	C10H20O2	000112-31-2	2197029	194255374
18	16.45	2,6,6-trimethyl-1-Cyclohexene-1-carboxaldehyde	C10H16O	000432-25-7	3972528	475174754
19	16.85	3,7-dimethyl-(Z)-2,6-Octadien-1-ol	C10H18O	000106-25-2	3260339	322821725
20	18.04	Geraniol	C10H18O	000106-24-1	14809938	1136559190
21	18.74	α-ethylidene-Benzeneacetaldehyde	C10H10O	004411-89-6	5903275	673980153
22	19.04	Nonanoic acid	C9H18O2	000112-05-0	4943616	1621642795
23	19.54	Eicosane	C20H42	000112-95-8	2008894	770208162
24	21.16	4-methyl-1-Undecene	C12H24	074630-39-0	3708390	274986102
25	21.97	1-Iodo-2-methylundecane	C12H25I	073105-67-6	1340762	91103122
26	22.21	1,2-dihydro-1,1,6-trimethyl-Naphthalene	C13H16	030364-38-6	766454	49167079
27	22.56	3,7-dimethyl-, (E)-2,6-Octadienoic acid	C10H16O2	004698-08-2	2364054	232108289
28	23.15	n-Decanoic acid	C10H20O2	000334-48-5	4813918	466893034

Number	Time	Compound name	Molecular formula	CAS	Peak height	Peak area
29	23.57	1-(2,6,6-trimethyl-1,3-cyclohexadien-1-yl)-, (E)-2-Buten-1-one	C13H18O	023726-93-4	2920271	183032388
30	24.16	ethyl ester-Decanoic acid	C12H24O2	000110-38-3	13104091	713079431
31	24.30	Nonadecane	C19H40	000629-92-5	2888502	208967030
32	24.65	Di-epi-α-cedrene	C15H24	050894-66-1	1557491	110972490
33	25.04	7-diol-2,4,7,9-Tetramethyl-5-decyn-4	C14H26O2	000126-86-3	4040516	240955051
34	25.36	α-Ionone	C13H20O	000127-41-3	3105890	168599927
35	26.09	2,6,6-trimethyl-1-Cyclohexene-1-butanol	C13H24O	054344-91-1	797510	38910373
36	26.44	6,10-dimethyl-(E)-5,9-Undecadien-2-one	C13H22O	003796-70-1	3458348	178024259
37	26.62	(E)-.β.-Farnesene	C15H24	018794-84-8	3543684	186280956
38	27.03	Undecanoic acid	C11H22O2	000112-37-8	1821981	85524078
39	27.18	5-dien-1-one-2,6-Di-tert-butyl-4-hydroxy-4-methylcyclohexa-2	C15H24O2	010396-80-2	1067175	66749660
40	27.32	(E)-2-Tetradecene	C14H28	035953-54-9	1229346	72525314
41	27.74	trans-.β.-Ionone	C13H20O	000079-77-6	18154259	910876440
42	27.89	5-Methyl-2-phenyl-2-hexenal	C13H16O	021834-92-4	2905935	186757067
43	28.05	(E)-5-Octadecene	C18H36	007206-21-5	1206236	43383638
44	28.18	Docosane	C22H46	000629-97-0	5809119	266736191
45	28.35	Pentadecane	C15H32	000629-62-9	3352079	253441258
46	28.82	2,5-bis(1,1-dimethylethyl)-Phenol	C14H22O	005875-45-6	3587486	202937417
47	29.28	5,6,7,7a-tetrahydro-4,4,7a-trimethyl-2(4H)-Benzofuranone	C11H16O2	015356-74-8	7675221	426586695
48	29.76	4-methyl-Heptadecane	C18H38	026429-11-8	975161	37979406
49	29.95	1-iodo-Tetracosane	C24H49I	1000406-32-0	3734818	264372578
50	30.21	Cyclopentadecane	C15H30	000295-48-7	917644	194306170
51	30.81	Nerolidol 2	C15H26O	1000285-43-6	13870894	595328395
52	31.11	Dodecanoic acid	C12H24O2	000143-07-7	9032247	1420254902
53	31.93	Cetene	C16H32	000629-73-2	1853473	113921738
54	32.22	Hexadecane	C16H34	000544-76-3	7243965	456098421
55	32.67	decyl-Oxirane	C12H24O	002855-19-8	1682152	160648090
56	33.1	Methyl 8-(2-furyl)octanoate	C13H20O3	038199-50-7	1968338	134755645
57	33.69	.tau.-Muurolol	C15H26O	019912-62-0	937422	50961293
58	34.83	3-methyl-Hexadecane	C17H36	006418-43-5	1146370	64562637
59	34.95	(Z)-3-Hexadecene	C16H32	034303-81-6	1131283	8959888
60	35.28	(4-Acetylphenyl)phenylmethane	C15H14O	000782-92-3	1118739	88396125
61	35.39	1-Octadecene	C18H36	000112-88-9	687836	62181787

Number	Time	Compound name	Molecular formula	CAS	Peak height	Peak area
62	35.41	Octacosyl heptafluorobutyrate	C32H57F7O2	1010351-83-6	684635	6096230
63	35.51	2-methylene-Cyclododecanone	C13H22O	003045-76-9	989156	57189316
64	35.65	1-Heptadecene	C17H34	006765-39-5	1022840	82516294
65	35.89	Heptadecane	C17H36	000629-78-7	3545352	200261141
66	36.56	butyl ester-6-Tetradecanesulfonic acid	C18H38O3S	1000280-27-4	957805	42879954
67	36.74	1-chloro-Nonadecane	C19H39Cl	062016-76-6	949990	27825967
68	36.82	Methyl tetradecanoate	C15H30O2	000124-10-7	1095351	70764701
69	37.67	Octadecane	C18H38	000593-45-3	1125270	206732988
70	37.78	ethyl octadecyl ether-1,3-Propanediol	C37H76O2	1000406-35-4	1409683	69864720
71	38.09	Tetradecanoic acid	C14H28O2	000544-63-8	1854505	133205190
72	38.32	3,5-Diethoxycarbonyl-2,6-dimethylpyr idine	C13H17NO4	001149-24-2	926231	30360872
73	38.94	(Z)-7-Hexadecene	C16H32	035507-09-6	647540	26743226
74	39.72	2,6,10,14-tetramethyl-Hexadecane	C20H42	000638-36-8	1240696	88172469
75	40.71	Caffeine	C8H10N4O2	000058-08-2	19434457	1022438877
76	40.92	6,10,14-trimethyl-2-Pentadecanone	C18H36O	000502-69-2	2585855	133061823
77	41.64	7-bromoheptyl isobutyl-esterPhthalic acid	C19H27BrO4	1000415-51-7	1498954	60653185
78	41.89	1-Nonadecene	C19H38	018435-45-5	509181	21207200
79	42.43	heptadecyl ester-Methoxyacetic acid-	C20H40O3	1000282-99-1	388616	3724553
80	43.28	(4,5)deca-6,9-diene-2,8-dione-7,9-Di-t ert-butyl-1-oxaspiro	C17H24O3	082304-66-3	1522011	105886494
81	43.61	methyl ester-Hexadecanoic acid	C17H34O2	000112-39-0	8136163	322192843
82	44.70	Dibutyl phthalate	C16H22O4	000084-74-2	3268180	132189632
83	44.80	n-Hexadecanoic acid	C16H32O2	000057-10-3	5651459	363732644
84	45.46	(Z)-9-Octadecenal	C18H34O	002423-10-1	421896	4900918
85	46.782	1-ethyl-5-(2-furanyl)-Pyrido[2,3-d]pyri midine-2,4(1H,3H)-dione	C13H11N3O3	1000362-82-6	336612	14108107
86	48.48	E-15-Heptadecenal	C17H32O	1000130-97-9	658655	34454407
87	48.80	methyl ester-9,12-Octadecadienoic acid	C19H34O2	002462-85-3	1623115	71256860
88	48.99	methyl ester-(Z,Z,Z)-9,12,15-Octadecatrienoic acid	C19H32O2	000301-00-8	2604685	116303402
89	49.36	Phytol	C20H40O	000150-86-7	6320121	276912324
90	49.83	14-methyl-Heptadecanoic acid	C19H38O2	002490-23-5	487085	25409148
91	51.56	butyl ester-Hexadecanoic acid	C20H40O2	000111-06-8	770311	28317396

Number	Time	Compound name	Molecular formula	CAS	Peak height	Peak area
92	54.69	2-chloro-1-(2,4-dichlorophenyl)-Ethanone	C8H5Cl3O	004252-78-2	318765	11879930

Table S5 Detailed information of the volatile compounds in zhutong tea (ZT).

Number	Time	Compound name	Molecular formula	CAS	Peak height	Peak area
1	6.14	Benzaldehyde	C7H6O	000100-52-7	1775827	592139004
2	6.75	Benzaldehyde	C7H6O	000100-52-7	1148133	592139004
3	8.57	D-Limonene	C10H16	005989-27-5	973035	34119171
4	9.09	Benzeneacetaldehyde	C8H8O	000122-78-1	1122121	118499608
5	9.32	1-ethyl-1H-Pyrrole-2-carboxaldehyde	C7H9NO	002167-14-8	1234497	205607838
6	11.40	Linalool	C10H18O	000078-70-6	7339691	691688303
7	11.90	Phenylethyl Alcohol	C8H8O	000060-12-8	2077762	318189478
8	14.03	diethyldimethyl-Plumbane	C6H16Pb	001762-27-2	4715556	803196460
9	14.69	Octanoic acid	C8H16O2	000124-07-2	1694369	215886430
10	15.25	α-Terpineol	C10H18O	000098-55-5	2561910	154698120
11	15.39	Methyl salicylate	C8H8O3	000119-36-8	3614283	464017995
12	15.65	Methyl salicylate	C8H8O3	000119-36-8	1567787	464017995
13	15.96	Decanal	C10H20O2	000112-31-2	723716	65487795
14	18.09	Geraniol	C10H18O	000106-24-1	3051551	202287483
15	19.15	Nonanoic acid	C9H18O2	000112-05-0	4465810	636342407
16	19.23	Eicosane	C20H42	000112-95-8	3391477	331147467
17	19.37	Anethole	C10H12O	000104-46-1	1145817	87769683
18	19.59	Eicosane	C20H42	000112-95-8	1013092	331147467
19	21.21	5-ethyl-2-methyl-Octane	C11H24	062016-18-6	2151124	128527611
20	22.02	Eicosyl nonyl ether	C29H60O	1000406-37-8	640611	38298559
21	22.22	1, 1, 5-Trimethyl-1, 2-dihydronaphthalene	C13H16	1000357-25-8	399457	28465956
22	23.06	n-Decanoic acid	C10H20O2	000334-48-5	1506284	141803306
23	23.607	1-(2,6,6-trimethyl-1,3-cyclohexadien-1-yl)-(E)-2-Buten-1-one	C13H18O	023726-93-4	537033	29073120
24	24.19	ethyl ester-Decanoic acid	C12H24O2	000110-38-3	16991771	820606421
25	24.326	Tetradecane	C14H30	000629-59-4	2683574	180802282
26	24.68	2,3,4,7,8,8a-hexahydro-3,6,8,8-tetramethyl-1H-3a,7-Methanoazulene	C15H24	000469-61-4	946212	64505164
27	25.051	7-diol-2,4,7,9-Tetramethyl-5-decyn-4	C14H26O2	000126-86-3	1499647	106955799
28	25.39	4-(2,6,6-trimethyl-2-cyclohexen-1-yl)-3-Bute n-2-one	C13H20O	006901-97-9	1076081	56355156
29	26.46	(E)- 6,10-dimethyl-5,9-Undecadien-2-one	C13H22O	003796-70-1	2073737	97634917
30	26.65	(1S,5S,6R)-6-Methyl-2-methylene-6-(4-meth ylpent-3-en-1-yl)bicyclo[3.1.1]heptane	C15H24	015438-94-5	659644	41576950
31	27.19	Pentadecane	C15H32	000629-62-9	569300	173274683

Number	Time	Compound name	Molecular formula	CAS	Peak height	Peak area
32	27.34	(Z)-3-Hexadecene	C16H32	034303-81-6	754634	45846035
33	27.65	4-(2,6,6-Trimethylcyclohexa-1,3-dienyl)but-3-en-2-one	C13H18O	001203-08-3	726798	29930478
34	27.75	β-Ionone	C13H20O	000079-77-6	5071132	239799799
35	27.99	Heptacosane	C27H56	000593-49-7	584476	136706756
36	28.07	1-Pentadecene	C15H30	013360-61-7	769654	34034437
37	28.20	Heptacosane	C27H56	000593-49-7	2587227	136706756
38	28.37	Pentadecane	C15H32	000629-62-9	3186661	173274683
39	28.84	2,4-Di-tert-butylphenol	C14H22O	000096-76-4	2217071	116239281
40	29.55	1-Iodo-2-methylundecane	C12H25I	073105-67-6	524582	28843947
41	29.97	1-iodo-Docosane	C22H45I	1000406-31-9	1474518	89644314
42	30.37	3,6-dimethyl-Decane	C12H26	017312-53-7	619418	52836246
43	31.07	Dodecanoic acid	C12H24O2	000143-07-7	7237742	646184900
44	31.94	Cetene	C16H32	000629-73-2	1121860	175757511
45	32.23	Hexadecane	C16H34	000544-76-3	6640288	301166514
46	32.67	Cetene	C16H32	000629-73-2	807862	175757511
47	34.04	2,6,10-trimethyl-Pentadecane	C18H38	003892-00-0	910596	56579966
48	34.163	Octadecanal	C18H36O	000638-66-4	894374	81363461
49	34.57	2-methyl-Hexadecane	C17H36	001560-92-5	789840	60540454
50	34.85	3-methyl-Hexadecane	C17H36	006418-43-5	601653	34534856
51	34.93	1-(ethenyloxy)-Decane	C12H24O2	000765-05-9	747532	34609007
52	35.41	N-[4-bromo-n-butyl]-2-Piperidinone	C9H16BrNO	195194-80-0	353728	7062264
53	35.65	1-Heptadecene	C17H34	006765-39-5	584247	43508943
54	35.91	Heptadecane	C17H36	000629-78-7	3250441	157892884
55	36.13	2,6,10,14-tetramethyl-Pentadecane	C19H40	001921-70-6	1247142	76183141
56	36.26	Tetracosane	C24H50	000646-31-1	886654	47795310
57	36.40	tridecyl-Oxirane	C15H30O	018633-25-5	744802	48060919
58	36.82	Cyclopentadecane	C15H30	000295-48-7	525732	35449960
59	37.72	Hexadecane	C16H34	000544-76-3	627298	301166514
60	37.79	Eicosane	C20H42	000112-95-8	673261	331147467
61	38.08	Tetradecanoic acid	C14H28O2	000544-63-8	921744	84568833
62	38.40	3,8-dimethyl-Decane	C12H26	017312-55-9	603012	68333325
63	38.64	E-15-Heptadecenal	C17H32O	1000130-97-9	444264	33419137
64	39.16	Cetene	C16H32	000629-73-2	553775	175757511
65	39.41	Octadecane	C18H38	000593-45-3	1713726	89328872
66	39.74	2,6,10,14-tetramethyl-Hexadecane	C20H42	000638-36-8	853089	60159405
67	39.95	Octadecanal	C18H36O	000638-66-4	465884	81363461
68	40.71	Caffeine	C8H10N4O2	000058-08-2	17915066	974737224

Number	Time	Compound name	Molecular formula	CAS	Peak height	Peak area
69	40.92	6,10,14-trimethyl-2-Pentadecanone	C18H36O	000502-69-2	2439045	106337449
70	41.41	Cyclotetradecane	C14H28	000295-17-0	406543	24216907
71	41.65	Di-sec-butyl phthalate	C16H22O4	1000373-65-4	904989	33715868
72	42.75	Nonadecane	C19H40	000629-92-5	541610	26039148
73	43.28	8-dione7-9-diene-2,9(4,5)deca-6,-Di-tert-butyl-1-oxaspiro	C17H24O3	082304-66-3	932040	53900664
74	43.61	methyl ester-Hexadecanoic acid	C17H34O2	000112-39-0	3321471	142293506
75	44.28	Isophytol	C20H40O	000505-32-8	793636	35270303
76	44.71	Dibutyl phthalate	C16H22O4	000084-74-2	2478928	93950524
77	44.78	n-Hexadecanoic acid	C16H32O2	000057-10-3	3848138	235901127
78	45.94	Eicosane	C20H42	000112-95-8	365106	331147467
79	47.94	N-[4-(1-methylethyl)benzylidene]-4-(1-pyrrolidylsulfonyl)-Benzenamine	C20H24N2O2S	314765-47-4	2705607	210099248
80	49.37	Phytol	C20H40O	000150-86-7	9876178	431096739
81	49.83	Methyl stearate	C19H38O2	000112-61-8	330700	13942083
82	51.56	butyl ester-Hexadecanoic acid	C20H40O2	000111-06-8	1418382	57893100

Table S6 Detailed information of the volatile compounds in green tea (GT).

Number	Time	Compound name	Molecular formula	CAS	Peak height	Peak area
1	6.98	*β*-Myrcene	C10H16	000123-35-3	1302101	7434431
2	11.21	Linalool	C10H18O	000078-70-6	11280858	1956120565
3	14.54	Terpinen-4-ol	C10H18O	000562-74-3	1127071	99568036
4	15.13	*α*-Terpineol	C10H18O	000098-55-5	937306	41984174
5	15.28	Methyl salicylate	C8H8O3	000119-36-8	1720589	252280011
6	15.88	Decanal	C10H20O2	000112-31-2	662209	41190614
7	16.47	2,6,6-trimethyl-1-Cyclohexene-1-carboxaldehyde	C10H16O	000432-25-7	852806	64618302
8	16.84	(Z)-3,7-dimethyl-,2,6-Octadien-1-ol	C10H18O	000106-25-2	499183	18475968
9	18.01	Geraniol	C10H18O	000106-24-1	2507143	145177909
10	18.86	Eicosane	C20H42	000112-95-8	864564	803575413
11	20.11	Pentacosane	C25H52	000629-99-2	1370509	123090334
12	21.16	2,3,6-trimethyl-Decane	C13H28	062238-12-4	3311125	176857065
13	21.87	4-methyl-Dodecane	C13H28	006117-97-1	730551	22318502
14	22.20	Nonadecane	C19H40	000629-92-5	345683	6826855
15	23.86	ethyl ester-3-ropenoic acid	C17H26NO5P	066564-08-7	10969037	523938536
16	24.16	ethyl ester-Decanoic acid	C12H24O2	000110-38-3	9278836	456464196
17	24.30	Tetradecane	C14H30	000629-59-4	1801374	130210001
18	25.36	*α*-Ionone	C13H20O	000127-41-3	804979	30426695
19	26.44	(E)-6,10-dimethyl-5,9-Undecadien-2-one	C13H22O	003796-70-1	985349	38943470
20	27.18	2,6-Di-tert-butyl-4-hydroxy-4-methylcyclohexa-2,5-dien-1-one	C15H24O2	010396-80-2	678314	27228240
21	27.31	methyl-Cyclooctane	C9H18	001502-38-1	569876	20332370
22	27.72	*trans*-*β*-Ionone	C13H20O	000079-77-6	4263435	189528187
23	27.97	2-ethyl-2-methyl-Tridecanol	C16H34O	1010115-66-1	938731	36720830
24	28.04	1-Heptadecene	C17H34	006765-39-5	713147	18868413
25	28.35	Pentadecane	C15H32	000629-62-9	1851572	84285278
26	28.82	2,4-Di-tert-butylphenol	C14H22O	000096-76-4	3447672	167296739
27	29.54	1-iodo-Docosane	C22H45I	1000406-31-9	935675	63382269
28	29.76	4-Methylheneicosane	C22H46	025117-29-7	767062	28578662
29	29.95	Heneicosane	C21H44	000629-94-7	3868955	338908471
30	30.34	2-Bromotetradecane	C14H29Br	074036-95-6	1245407	76128043
31	31.21	Hexadecane	C16H34	000544-76-3	911211	197229444
32	31.59	1-iodo-Dodecane	C12H25I	004292-19-7	821564	31373337
33	31.93	(Z)-7-Hexadecene	C16H32	035507-09-6	924671	42028449

Number	Time	Compound name	Molecular formula	CAS	Peak height	Peak area
34	32.12	2,2,4-Trimethyl-1,3-pentanediol diisobutyrate	C16H30O4	006846-50-0	1686678	74543238
35	32.66	Cyclododecanol	C12H24O2	001724-39-6	665699	30228544
36	33.67	α-Cadinol	C15H26O	000481-34-5	386515	15304179
37	34.04	2,6,10,14-tetramethyl-Nonadecane	C23H48	055124-80-6	623085	29294787
38	34.56	2-methyl-Hexadecane	C17H36	001560-92-5	499418	23315216
39	35.64	Z-8-Hexadecene	C16H32	1000130-87-5	463655	19487235
40	35.89	Heptadecane	C17H36	000629-78-7	1946555	93766336
41	36.12	2,6,10,14-tetramethyl-Hexadecane	C20H42	000638-36-8	1261022	94120808
42	36.38	Tetradecanal	C11H20O2	000124-25-4	657991	25760207
43	37.79	Hentriacontane	C31H64	000630-04-6	1690250	92742638
44	38.32	3,5-Diethoxycarbonyl-2,6-dimethylpyridine	C13H17NO4	001149-24-2	486028	12588720
45	39.40	Octadecane	C18H38	000593-45-3	1197667	52564669
46	40.66	Caffeine	C8H10N4O2	000058-08-2	3928943	144556183
47	40.72	Neophytadiene	C20H38	000504-96-1	6080199	242517201
48	40.92	6,10,14-trimethyl-2-Pentadecanone	C18H36O	000502-69-2	1530198	69615866
49	41.64	isobutyl undecyl ester-Phthalic acid	C23H36O4	1010308-97-3	674163	20679140
50	43.61	methyl ester-Hexadecanoic acid	C17H34O2	000112-39-0	3877243	149091771
51	44.74	n-Hexadecanoic acid	C16H32O2	000057-10-3	1712913	111887254
52	48.80	(Z,Z)-methyl ester-9,12-Octadecadienoic acid	C19H34O2	000112-63-0	536039	21474995
53	48.99	(Z,Z,Z)-methyl ester-9,12,15-Octadecatrienoic acid	C19H32O2	000301-00-8	1603533	66036354
54	49.36	Phytol	C20H40O	000150-86-7	9008065	368208232
55	49.83	Methyl stearate	C19H38O2	000112-61-8	660268	24159718
56	51.56	butyl ester-Hexadecanoic acid	C20H40O2	000111-06-8	750626	28363382
57	54.70	Ethyl 3-[1-(2,6-dichlorobenzoyl)pyrrol-2-yl]prop-2-enoate	C16H13Cl2NO3	095883-17-3	300663	12712001
